# Prolactin in man: a tale of two promoters

**DOI:** 10.1002/bies.20468

**Published:** 2006-10

**Authors:** Sarah Gerlo, Julian RE Davis, Dixie L Mager, Ron Kooijman

**Affiliations:** 1 Laboratory of Eukaryotic Gene Expression and Signal Transduction, Department of Molecular Biology, Ghent UniversityBelgium; 2 Endocrine Sciences Research Group, Faculty of Medical and Human Sciences, University of ManchesterUK; 3 Terry Fox Laboratory, British Columbia Cancer AgencyCanada; 4 Laboratory of Neuroendocrine Immunology, Free University of BrusselsBelgium

## Abstract

The pituitary hormone prolactin (PRL) is best known for its role in the regulation of lactation. Recent evidence furthermore indicates PRL is required for normal reproduction in rodents. Here, we report on the insertion of two transposon-like DNA sequences in the human prolactin gene, which together function as an alternative promoter directing extrapituitary PRL expression. Indeed, the transposable elements contain transcription factor binding sites that have been shown to mediate PRL transcription in human uterine decidualised endometrial cells and lymphocytes. We hypothesize that the transposon insertion event has resulted in divergent (pituitary versus extrapituitary) expression of prolactin in primates, and in differential actions of pituitary versus extrapituitary prolactin in lactation versus pregnancy respectively. Importantly, the TE insertion might provide a context for some of the conflicting results obtained in studies of PRL function in mice and man. BioEssays 28: 1051–1055, 2006. © 2006 Wiley Periodicals, Inc.

## Introduction

**Figure 1:**
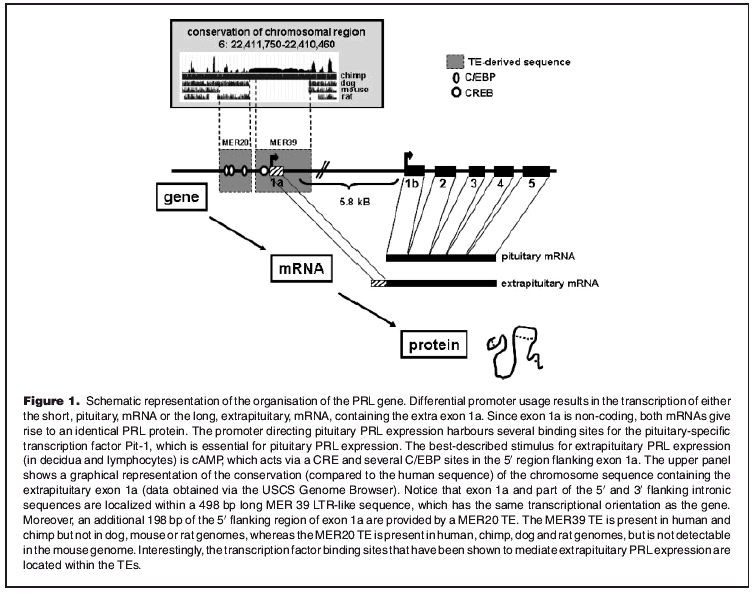
Schematic representation of the organisation of the PRL gene. Differential promoter usage results in the transcription of either the short, pituitary, mRNA or the long, extrapituitary, mRNA, containing the extra exon 1a. Since exon 1a is non–coding, both mRNAs give rise to an identical PRL protein. The promoter directing pituitary PRL expression harbours several binding sites for the pituitary^–specific transcription factor Pit–1, which is essential for pituitary PRL expression. The best–described stimulus for extrapituitary PRL expression (in decidua and lymphocytes) is cAMP, which acts via a CRE and several C/EBP sites in the 5′ region flanking exon 1a. The upper panel shows a graphical representation of the conservation (compared to the human sequence) of the chromosome sequence containing the extrapituitary exon 1a (data obtained via the USCS Genome Browser). Notice that exon 1a and part of the 5′ and 3′ flanking intronic sequences are localized within a 498 bp long MER 39 LTR–like sequence, which has the same transcriptional orientation as the gene. Moreover, an additional 198 bp of the 5′ flanking region of exon 1a are provided by a MER20 TE. The MER39 TE is present in human and chimp but not in dog, mouse or rat genomes, whereas theMER20TEis present in human, chimp, dog and rat genomes, but is not detectable in the mouse genome. Interestingly, the transcription factor binding sites that have been shown to mediate extrapituitary PRLexpression are located within the TEs.

The polypeptide hormone prolactin (PRL) is produced mainly by the lactotrope cells of the anterior pituitary and, in mammals, its most apparent function is the regulation of lactation.(1) In addition, PRL has been attributed an important role in reproduction, as was illustrated by the observation that PRL^−/−^ mice not only have defects in mammopoiesis but are also sterile.(2) Interestingly, although the bulk ofPRLcirculating in serum is produced by the lactotrope cells of the pituitary, PRL is also expressed extrapituitarily by various tissues including uterine decidualised endometrial cells and leukocytes.(3) The human PRL gene is located on chromosome 6 and was initially described as containing five exons and four introns, with an overall length of 10 kilobases (kB).(4) However, when extrapituitary PRL expression was shown in human lymphocytes and subsequently in human decidual cells, it was revealed that, in these extrapituitary tissues, the PRL messenger RNA (mRNA) was longer than its pituitary counterpart.(5,6) The reason for this discrepancy is that, in human decidua and lymphocytes, PRL transcription is driven by an alternative promoter, which is located 5.8 kb upstream to the pituitary transcription start site, resulting in the transcription of an extra exon, named exon 1a (Fig. 1).(7,8)

Alternative promoter usage is not an uncommon phenomenon. Landry et al, by screening databases for transcripts with alternative first exons, estimated that approximately 18% of human genes have alternative promoters.(9) According to a more recent paper, the rate of alternative promoter usage is even higher and approximately half of the human genome was predicted to be transcribed via multiple promoters.(10) In the case of the PRL gene, the alternative initial exon does not contain coding sequence and hence transcription from the alternative promoter does not lead to the generation of protein isoforms. However, the extra exon could contribute to the expansion of the transcriptional and/or translational “repertoire” of the PRL gene. Indeed, alternative promoters often evolve in genes with complex patterns of transcriptional regulation. For instance, there are many examples of genes where an alternative promoter confers tissue–specific expression, directs expression as a function of the differentiation stage and/or regulates expression level.(11–15) In addition, the variant 5′ untranslated regions (UTRs) might influence the stability or translational efficiency of the alternatively promoted messages.(16–18)

## Two transposable elements (TEs) direct extrapituitary PRL expression

To date, the use of an alternative promoter to regulate extrapituitary PRL expression has only been described in humans and in the rhesus macaque.(7,8, 19) Intriguingly, by screening the PRL gene using the UCSC Genome Browser and its RepeatMasker facility (http://genome.ucsc.edu/cgi-bin/hgGateway), we observed that the proximal part of the extrapituitary PRL promoter, as well as exon 1a and a part of intronic sequence (Chr6: 22,410,692–22,411,189=498 bp), occur within a long terminal repeat (LTR)–like transposable element (TE) of the medium frequency reiterated repeat (MER) family.(20) The LTR sequence (named MER39) belongs to the ERV1 class of endogenous retroviruses and it is also present in the Rhesus macaque and chimpanzee PRL gene, but not in dog, mouse or rat genomes (Fig. 1). This indicates that insertion of the LTR element occurred at least 25–30 million years ago, before the divergence of Old World monkeys from higher apes. In addition, an older TE, MER20, belonging to the MER1 class of DNA transposons, has provided the sequence between −189 and −387 of the extrapituitary PRL promoter (Chr6: 22,411,225–22,411,422=198 bp). This TE is also present in the rat and dog, but is not evident in mice (Fig. 1). It is likely that the MER20 TE integrated before mammalian radiation, 70–80 million years ago, but is not found by Repeatmasker in mice due to the higher neutral mutation rate in rodents, making ancient TEs difficult to detect.(21) Several reports have demonstrated that TE–derived sequences can regulate nearby human genes. In the majority of these cases, the LTR acts as a gene promoter, is often one of multiple alternative promoters and usually does not alter the coding sequence.(14,15,22–24)

PRL transcription via the extrapituitary PRL promoter has been most intensively studied in decidual cells and in lymphocytes. In both cell types, promoter activity is stimulated by cAMP and, interestingly, the responsiveness to cAMP is in part mediated by an imperfect cAMP–responsive element (CRE), located within the MER39 LTR (Fig. 1).(25,26) Moreover, two CAAT/enhancer binding protein (C/EBP) sites in the neighbouring 5′ promoter sequence, which is derived from a MER20 transposon, have also been shown to be obligate for cAMP responsiveness both in decidual cells and in lymphocytes, suggesting the two TEs have acquired the ability to cooperate to promote extrapituitary PRL expression in primates.(27,28)

## In search of a role for primate extrapituitary PRL

To our knowledge, there are no reports of mutations in the PRL gene in humans. This could mean that such mutations do not have any important phenotypic consequences, or, alternatively, that they are lethal. PRL deficiency has been described in individuals with mutations in the Pit–1 transcription factor, which is essential for transcription of pituitary PRL and other pituitary hormones.(29) However, extrapituitary PRL expression does not depend on Pit–1 and hence is probably not affected in these subjects. In addition, few cases of women with isolated PRL deficiency have been reported that became apparent only because these women suffered from alactogenesis (after normal pregnancies).(30–33) In these case studies, the involvement of a genetic defect in the observed PRL deficiency was not addressed. Importantly, in the above studies, PRL deficiency was defined as undetectable PRL levels in serum and there is no evidence that there was in fact no local, extrapituitary, PRL production. The assays that are routinely used in the clinic to detect PRL in serum typically have detection limits of around 500 pg/ml, which is adequate for detection of endocrine PRL, but probably not for detection of autocrine or paracrine PRL, that is secreted extrapituitarily. Therefore, to establish whether these “PRL–deficient” individuals are truly PRL knock–outs, extrapituitary PRL expression should also be assessed.

Whereas women with isolated PRL deficiency, as demonstrated by undetectable serum PRL levels, can reproduce normally but do not lactate, a recent paper describes a correlation between defective decidual PRL expression and implantation failure, suggesting extrapituitary PRL might play a role in the regulation of reproduction.(34) An intriguing question is whether alternative promoter usage, due to TE insertions, has led to different functions for pituitary and extrapituitary PRL, i.e. in lactation and pregnancy, respectively. It is conceivable that bifurcated PRL expression is energetically favourable and perhaps advantageous in an evolutionary perspective, because high PRL levels in the amniotic fluid required for successful reproduction are delivered by the decidua, and hence secretion of large amounts of PRL by the pituitary can be limited to the period of lactation. An interesting parallel can be drawn with the case of the leptin gene. Leptin is a circulating hormone that is expressed by adipose tissue and is involved in the regulation of energy homeostasis, as well as neuroendocrine and reproductive systems. In mice, serum leptin levels increase dramatically during pregnancy. In man there is only a moderate increase in serum leptin levels, yet during pregnancy, leptin is produced by the placenta and accumulates in the amniotic fluid. Interestingly, it has been shown that placental leptin expression in humans is also driven by a primate/specific TE inserted in the human leptin gene.(35)

PRL^−/−^ mice, which are infertile, lack pituitary as well as extrapituitary PRL, so it is possible that, in mice also, extrapituitary (decidual) PRL plays a role in reproduction. However, whereas there is ample evidence for extrapituitary PRL expression in rodents and other non-primates, the PRL mRNAs detected in these species have not been characterized in detail.(3) Unless evidence is provided for the transcription of PRL mRNAs with alternative first exons, it is most likely that the described PRL transcripts are under the control of the same promoter that is active in the pituitary. Of course, tissuespecific functions of PRL may still exist independently of alternative promoter usage in non–primates.

Addition of an alternative promoter through TE insertion in primates may also have led to specialized functions of PRL in the human immune system. A point of controversy is that there are many indications for a role of PRL in the human immune system, whereas PRL or PRL–R knockout mice are not immune deficient.(2,36) It could be that, in primates, local PRL, produced in the immune system under the control of the alternative upstream promoter has a special, cytokine–like, function. Interestingly, in species, such as the mouse, the rat and the cow, but not in primates, the PRL gene has expanded to yield a large family of paralogous genes closely related to PRL. Mice possess at least 26 paralogous PRL genes and current information indicates that not all members of this PRL family use the PRL receptor.(37) Neither knocking out PRL nor the PRL–R would therefore create a model system mimicking the situation of a human PRL knockout. One could speculate that the lack of expansion of the PRL gene in primates is compensated by the mechanism of alternative promoter usage and that, in primates, certain functions of PRL or PRL–like proteins are exerted by extrapituitary PRL under the control of the alternative promoter.

## Perspectives

In summary, we believe further research should be directed towards elucidating the consequences and the importance of the primate–specific organization of the PRL gene. As has been shown for other examples of TEs acting as gene promoters, it would be interesting to determine if the TEs involved in PRL expression possessed the CREB site and other regulatory motifs at the time of insertion and if these motifs have been selectively conserved throughout primate evolution.(38) If so, it would argue for an important role for extrapituitary PRL expression. In view of the recent observation that there is a relationship between decidual PRL expression and reproductive disorders, further research should be directed towards assessing the molecular basis of the reported defects in extrapituitary PRL expression and the consequences of these defects in reproduction. In addition, it would be interesting to evaluate extrapituitary PRL expression in women with isolated prolactin deficiency. If our hypothesis that extrapituitary PRL is involved in reproduction is correct, then one would expect to detect PRL in decidual cells from these women.

Unfortunately, whereas the mouse model has been an invaluable tool to study human biology and disease, data regarding extrapituitary PRL expression cannot be readily extrapolated from mice to man. Rodent transgenic knock–in approaches could be useful though to study the function of the alternative promoter in vivo. Transgenes of interest could therefore include either the whole hPRL locus, or the pituitary promoter alone or the alternative promoter alone, either in normal animals or in PRL^−/−^ mice. This approach would allow for an unambiguous evaluation of the contribution of the two PRL promoters to tissue–specific PRL expression. Additionally, if our speculation is right, one would expect that knock–in of hPRL expression from the extrapituitary promoter alone would be sufficient to rescue PRL^−/−^ mice from infertility. However, as it is unlikely that human reproduction can be mimicked entirely in mice, primate (for instance Rhesus monkey) models will be indispensable to gain a better insight into the role of human extrapituitary PRL expression.

## Abbreviations:

PRL: prolactin
TE: transposable element
kB: kilobase
mRNA: messenger RNA
UTR: untranslated region
LTR: long terminal repeat
MER: medium reiterated frequency repeat
CRE: cAMP-responsive element
C/EBP: CAAT enhancer-binding protein
